# Dysfunction of intraflagellar transport-A causes hyperphagia-induced obesity and metabolic syndrome

**DOI:** 10.1242/dmm.025791

**Published:** 2016-07-01

**Authors:** Damon T. Jacobs, Luciane M. Silva, Bailey A. Allard, Michael P. Schonfeld, Anindita Chatterjee, George C. Talbott, David R. Beier, Pamela V. Tran

**Affiliations:** 1 Department of Anatomy and Cell Biology andThe Kidney Institute, University of Kansas Medical Center, Kansas City, KS 66160, USA; 2 Genetics Division, Brigham and Women's Hospital, Harvard Medical School, Boston, MA 02115, USA; 3 Center for Developmental Biology and Regenerative Medicine, Seattle Children's Research Institute, Seattle, WA 98105, USA

**Keywords:** Primary cilia, IFT complex A, POMC, Obesity mouse model

## Abstract

Primary cilia extend from the plasma membrane of most vertebrate cells and mediate signaling pathways. Ciliary dysfunction underlies ciliopathies, which are genetic syndromes that manifest multiple clinical features, including renal cystic disease and obesity. *THM1* (also termed *TTC21B* or *IFT139*) encodes a component of the intraflagellar transport-A complex and mutations in *THM1* have been identified in 5% of individuals with ciliopathies. Consistent with this, deletion of murine *Thm1* during late embryonic development results in cystic kidney disease. Here, we report that deletion of murine *Thm1* during adulthood results in obesity, diabetes, hypertension and fatty liver disease, with gender differences in susceptibility to weight gain and metabolic dysfunction. Pair-feeding of *Thm1* conditional knock-out mice relative to control littermates prevented the obesity and related disorders, indicating that hyperphagia caused the obese phenotype. *Thm1* ablation resulted in increased localization of adenylyl cyclase III in primary cilia that were shortened, with bulbous distal tips on neurons of the hypothalamic arcuate nucleus, an integrative center for signals that regulate feeding and activity. In pre-obese *Thm1* conditional knock-out mice, expression of anorexogenic *pro-opiomelanocortin* (*Pomc*) was decreased by 50% in the arcuate nucleus, which likely caused the hyperphagia. Fasting of *Thm1* conditional knock-out mice did not alter *Pomc* nor orexogenic *agouti-related neuropeptide* (*Agrp*) expression, suggesting impaired sensing of changes in peripheral signals. Together, these data indicate that the *Thm1*-mutant ciliary defect diminishes sensitivity to feeding signals, which alters appetite regulation and leads to hyperphagia, obesity and metabolic disease.

## Abstract (precis)

**Summary:** Disruption of the IFT-A complex gene, *Thm1*, in adult mice misregulates response to feeding signals, altering appetite regulation and resulting in obesity through hyperphagia.

## INTRODUCTION

Obesity is a global epidemic with significant morbidity and mortality. Obesity often leads to metabolic syndrome, a combination of adverse health conditions that includes dyslipidemia, hypertension, glucose intolerance and insulin resistance (O'Neill and O'Driscoll, 2014). These increase risk for diabetes mellitus type 2, cardiovascular disease and non-alcoholic fatty liver disease, for which treatments are invasive and largely ineffective (Shin et al., 2013). Despite extensive investigations, much is still unknown regarding the molecular mechanisms underlying onset of obesity and associated metabolic disorders.

Obesity arises when caloric intake exceeds caloric expenditure. This energy balance is controlled by neural circuitry that initiates in the hypothalamic arcuate nucleus (ARC), a central processing center for signals that regulate feeding and activity. In the ARC, neurons expressing pro-opiomelanocortin (POMC) and agouti-related peptide/neuropeptide Y (AgRP/NPY) are two distinct neuron populations that respond to signals emanating from peripheral tissues (Sohn et al., 2013). In response to feeding, satiety signals, such as leptin or insulin, are released into the bloodstream by adipose tissue and the pancreas. Upon reaching the POMC-expressing neurons of the ARC, these elicit a response to stop food-seeking behavior and increase physical activity (Millington, 2007). In contrast, fasting signals such as ghrelin, which is secreted by an empty stomach, signal to the AgRP/NPY-expressing neurons to elicit a food-seeking response (Liu et al., 2012). In the satiated state, leptin further inhibits AgRP/NPY-expressing neurons to enhance the satiety signal. Deficiency of leptin or of the leptin receptor in the *ob/ob* or *db/db* mouse models, respectively, dysregulates the feeding/activity signaling axis resulting in excessive food intake (hyperphagia) and obesity (Islam, 2013).

Ciliopathies are genetic syndromes that link hyperphagia and obesity to dysfunction of the primary cilium, an antenna-like sensory organelle that regulates signaling pathways and is present on almost all vertebrate cells (Berbari et al., 2009). Within the cilium, protein complexes carry cargoes of structural or signaling proteins bidirectionally along microtubular tracks in a process termed intraflagellar transport (IFT). The IFT machinery comprises IFT-B and IFT-A protein complexes, which are transported by kinesin and cytoplasmic dynein motors. Ciliopathies affect multiple organs and clinical features can include cystic disease of the kidney, liver and pancreas, retinal degeneration, facial anomalies, mental retardation and polydactyly. In two ciliopathies, Bardet–Beidl syndrome (BBS) and Alström syndrome, obesity also presents as a major clinical feature (Girard and Petrovsky, 2011; Quinlan et al., 2008). BBS results from mutations of at least 20 genes (Lindstrand et al., 2014), which encode products that facilitate or assemble into a protein complex, the BBSome, which transports cargo to membrane compartments and within the ciliary membrane (Guo and Rahmouni, 2011). Alström syndrome results from mutations of a single gene, *ALMS1*, whose gene product localizes to the basal body (Girard and Petrovsky, 2011). In mice, loss of *Bbs2*, *Bbs4* and *Bbs6* causes hyperphagia, obesity and hyperleptinemia (Rahmouni et al., 2008), and hypothalamic neurons of *Bbs4^−/−^* mice lack ciliary localization of appetite-regulating G-protein coupled receptors (Berbari et al., 2008). *Alms1*-mutant mice are hyperphagic and obese (Arsov et al., 2006; Collin et al., 2005), and *foz/foz* mice, which harbor a truncating mutation in *Alms1*, manifest a progressive loss of neuronal cilia in the hypothalamus (Heydet et al., 2013). Further, ablation of the complex B genes, *Ift88* or *Kif3a*, either during adulthood or specifically in POMC-expressing cells of mice, causes hyperphagia, obesity and hyperleptinemia (Davenport et al., 2007). These data underscore the importance of neuronal primary cilia in regulating the feeding/activity signaling axis. Finally, hypomorphic *Rpgrip1l*^+/−^ mutants of the transition zone, which regulates entry of proteins into the cilium, are also hyperphagic and obese (Stratigopoulos et al., 2014).

A role for IFT complex A in regulating energy homeostasis has not been reported. Previously, we identified THM1 as an IFT complex A protein (Tran et al., 2008), and pathogenic alleles of *THM1* have been identified in 5% of individuals with ciliopathies, including BBS, for which obesity is a major clinical component (Davis et al., 2011). Loss of THM1 impairs retrograde IFT, causing shortened primary cilia with bulbous distal tips where protein particles accumulate (Tran et al., 2008). In mouse, early embryonic loss of *Thm1* causes polydactyly, craniofacial and neural tube defects and perinatal lethality (Tran et al., 2008), whereas deletion of *Thm1* during late embryogenesis causes cystic kidney disease (Tran et al., 2014). Collectively, these mouse mutants demonstrate many of the clinical features of ciliopathies. Because obesity is a primary clinical feature of BBS, we examined whether deletion of murine *Thm1* also causes obesity and affects neuronal signaling in the ARC, misregulating energy homeostasis.

## RESULTS

### *Thm1* conditional knock-out mice become obese

**Fig. 1.**
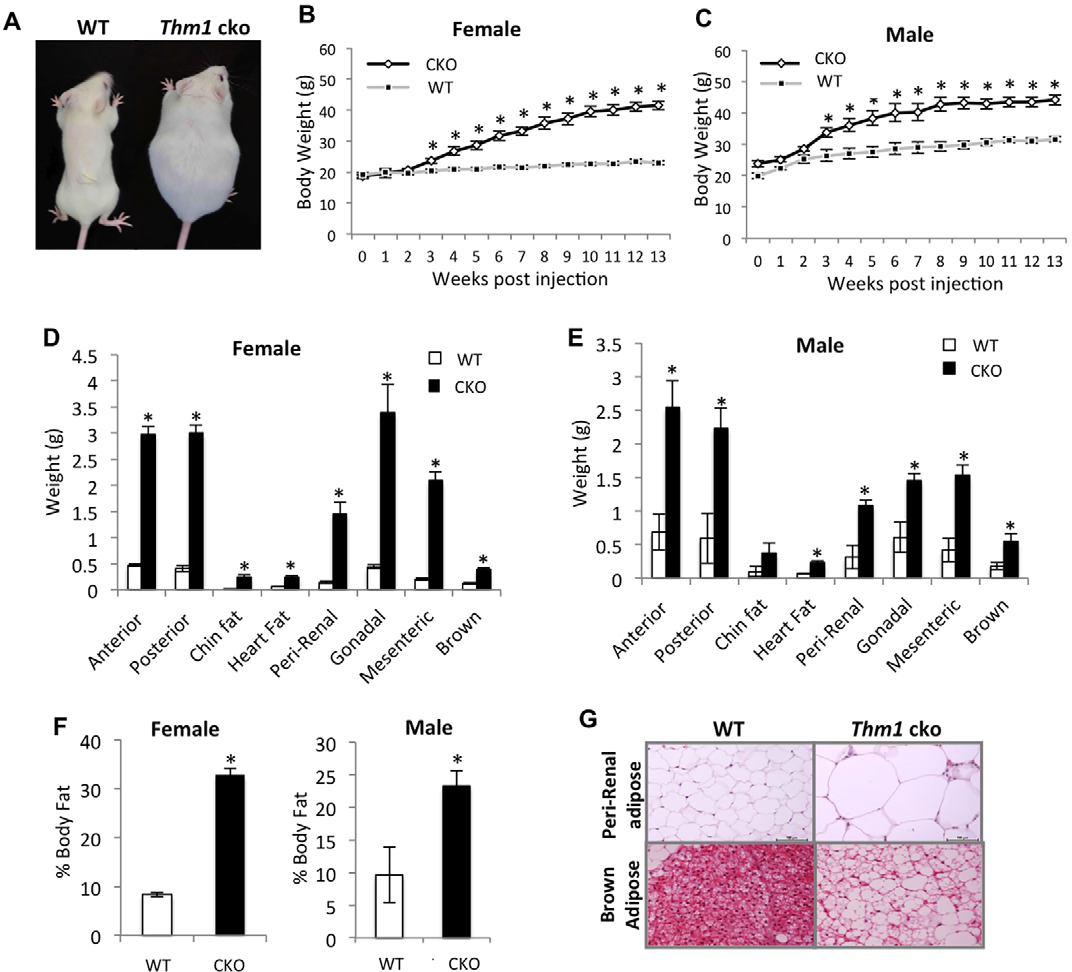
**Loss of *Thm1* causes obesity.** (A) *Ad libitum*-fed WT and *Thm1*-cko mice 13 weeks post-tamoxifen injection. A conditional allele of *Thm1* was deleted using a tamoxifen-inducible Cre recombinase at 5 weeks of age. (B,C) Weekly body weight measurements over a 13-week period. Data points represent means±s.e.m. Two-tailed unequal variance *t*-test. *P<0.05. *N*=9 WT; *N*=9 *Thm1*-cko each for male and female. (D,E) Adipose tissue weights at 13 weeks post-tamoxifen injection. Error bars represent means±s.e.m. *P<0.05. (F) Percent body fat. Isolated adipose depots were weighed and summed, then divided over body weight. Error bars represent means±s.e.m. *P<0.05. (G) H&E staining of peri-renal fat and brown fat. Scale bars: 100 µm.

We deleted *Thm1* at 5 weeks of age using a tamoxifen-inducible Cre recombinase driven by the *Rosa26* locus, then monitored *Thm1* conditional knock-out (cko) mice over a 13-week period. Three weeks following gene deletion, *Thm1*-cko mice showed significantly increased body weight relative to wild-type control (WT) littermates. By thirteen weeks post-*Thm1* ablation, *Thm1*-cko females and males showed 1.8- and 1.4-fold higher body weights, respectively than their WT littermates (Fig. 1A-C). Adipose depots of *Thm1*-cko mice were significantly larger (Fig. S1; Fig. 1D,E), and *Thm1*-cko females and males exhibited ∼3.7- and 2.0-fold higher percentage body fat than female and male WT littermates, respectively (Fig. 1F). Histological analysis of peri-renal, white adipose tissue revealed that *Thm1*-cko adipocytes were enlarged ∼threefold in diameter, corresponding to a 45-fold increase in volume (Fig. 1G, upper panels). Analysis of WT subcutaneous scapular brown adipose tissue (BAT) showed numerous small locules containing lipid droplets. In contrast, *Thm1*-cko BAT showed loss of lipid locules and increased lipid droplet size, characteristic of obesity (Fig. 1G, lower panels).

To confirm genomic recombination of *Thm1*, mice harboring a tdTomato;EGFP reporter, which fluoresces tdTomato in non-recombined tissue and EGFP in recombined tissue, were bred into the *Thm1^fl/fl^* line, which was subsequently crossed to *Thm1^aln/+^;Cre+* mice. The *aln* allele is a null allele. Fluorescence analysis of arcuate nucleus, hypothalamus, skeletal muscle and white and brown adipose tissues of progeny that were tamoxifen-injected and harboring a *Cre*+ allele fluoresced green, indicating recombination (Fig. S2). Varying expression of tdTomato was also observed in these tissues, indicating recombination was partial. Skeletal muscle showed the most effective recombination. Western blot analysis was also performed on protein extracts of the arcuate nucleus, hypothalamus, skeletal muscle and white and brown adipose tissues. *Thm1*-cko tissues showed 35-45% less protein relative to control *Thm1^fl/+^;Cre+* extracts (Fig. S2).

### *Thm1*-cko mice develop metabolic syndrome

**Fig. 2.**
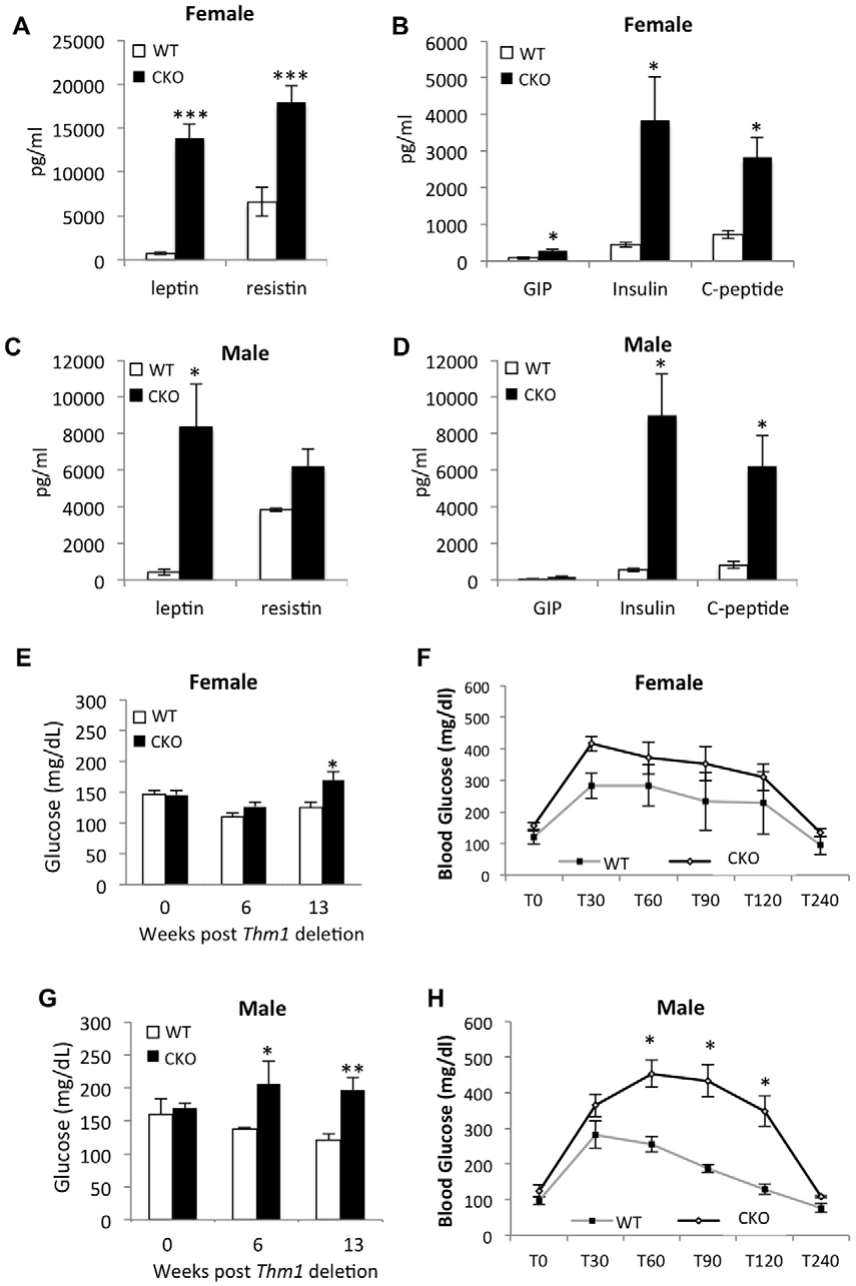
**Obese *Thm1*-cko mice show hyperleptinemia, hyperinsulinemia and diabetes.** (A-D) Serum metabolite levels at 13 weeks post-tamoxifen injection. (E,G) Blood glucose levels at 0, 6 and 13 weeks post-tamoxifen injection. *N*=6 WT; *N*=6 *Thm1*-cko each for male and female. (F,H) Glucose tolerance test. Mice ranging from 13 to 20 weeks post-*Thm1* deletion were challenged with a glucose bolus (2 mg/g body weight) at T0 by i.p. injection. Blood glucose was monitored at 30-min intervals to determine clearance rate. *N*=3 WT females; *N*=6 *Thm1*-cko females; *N*=4 WT males; *N*=6 *Thm1*-cko males. Data points represent means±s.e.m. Two-tailed unequal variance *t*-test. *P<0.05.

To determine whether the *Thm1*-cko obese phenotype was accompanied by metabolic abnormalities, we measured serum levels of adipocyte-derived leptin and resistin, pancreatic β-cell-derived insulin and C-peptide, and glucose-dependent insulinotropic peptide (GIP) in WT and *Thm1*-cko mice at 13 weeks post-*Thm1* deletion using a metabolic multiplex ELISA. Resistin has been linked to diabetes, cardiovascular disease and non-alcoholic fatty liver disease and is proposed to modulate metabolic and inflammatory pathways (Jamaluddin et al., 2012). Insulin promotes uptake of blood glucose into skeletal muscle, adipose tissue and liver, and like leptin, decreases appetite in the hypothalamus. Upon insulin formation, C-peptide is released as a by-product, and thus, C-peptide levels often reflect levels of insulin synthesis (Landreh et al., 2013). Finally, GIP is released by cells of the gastrointestinal tract and stimulates insulin secretion in response to glucose (Lynn et al., 2001). Leptin, insulin and C-peptide levels were elevated in both *Thm1*-cko males and females, and resistin and GIP levels were also increased in *Thm1*-cko females (Fig. 2A-D). These data indicate disturbances in insulin metabolism and resistance to leptin and insulin in obese *Thm1*-cko mice.

To examine glucose and insulin metabolism further, we measured blood glucose levels at 0, 6 and 13 weeks post-tamoxifen injection. At 6 and 13 weeks post-tamoxifen injection, *Thm1*-cko mice showed elevated glucose levels relative to WT mice (Fig. 2E,G). We next performed a glucose tolerance test (GTT), which measures ability to clear a glucose load from the bloodstream. An altered response to a GTT can serve as an indicator of diabetes mellitus type 2 (DM2), which is characterized by an inability to effectively and efficiently transfer glucose from the bloodstream into tissues via insulin signaling (Muoio and Newgard, 2008). Following a bolus intraperitoneal (i.p.) injection of a 20% glucose solution at time 0 (T0), blood glucose spiked at 30 min (T30) and gradually cleared to fasting levels by 2 h (T120) in WT and *Thm1*-cko mice (Fig. 2F,H). *Thm1*-cko females showed a similar glucose clearance as WT females. However, in *Thm1*-cko males, glucose levels continued to rise until 1 hour post-injection (T60) and remained significantly elevated at 2 h (T120) (Fig. 2H). This impaired response suggests a diabetic state in *Thm1*-cko males.

**Fig. 3.**
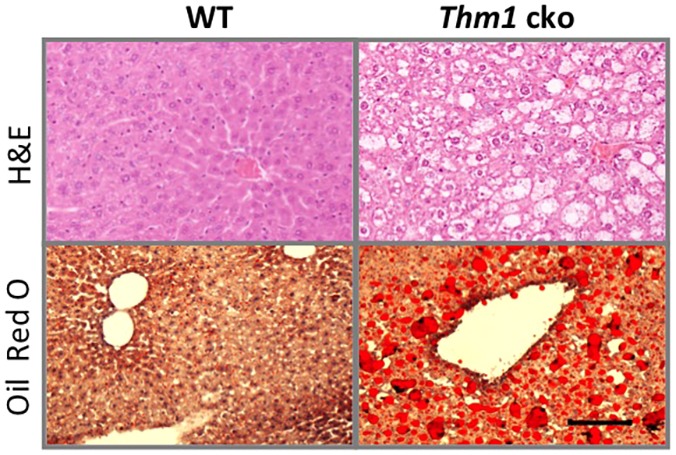
**Obese *Thm1*-cko mice show hepatic steatosis.** H&E and Oil Red O staining of liver sections from WT and *Thm1*-cko mice at 13 weeks post-tamoxifen injection. Scale bar: 100 µm.

In addition, we weighed internal organs and found that the livers of *Thm1*-cko mice were significantly heavier than those of WT mice (Fig. S3). Fatty deposits were visible on *Thm1*-cko livers at the whole-mount level (data not shown), and histological analysis of liver sections using H&E and Oil Red O staining confirmed a marked increase in size and number of lipid droplets in *Thm1*-cko liver, indicative of fatty liver disease (Fig. 3).

### *Thm1*-cko mice are hyperphagic

**Fig. 4.**
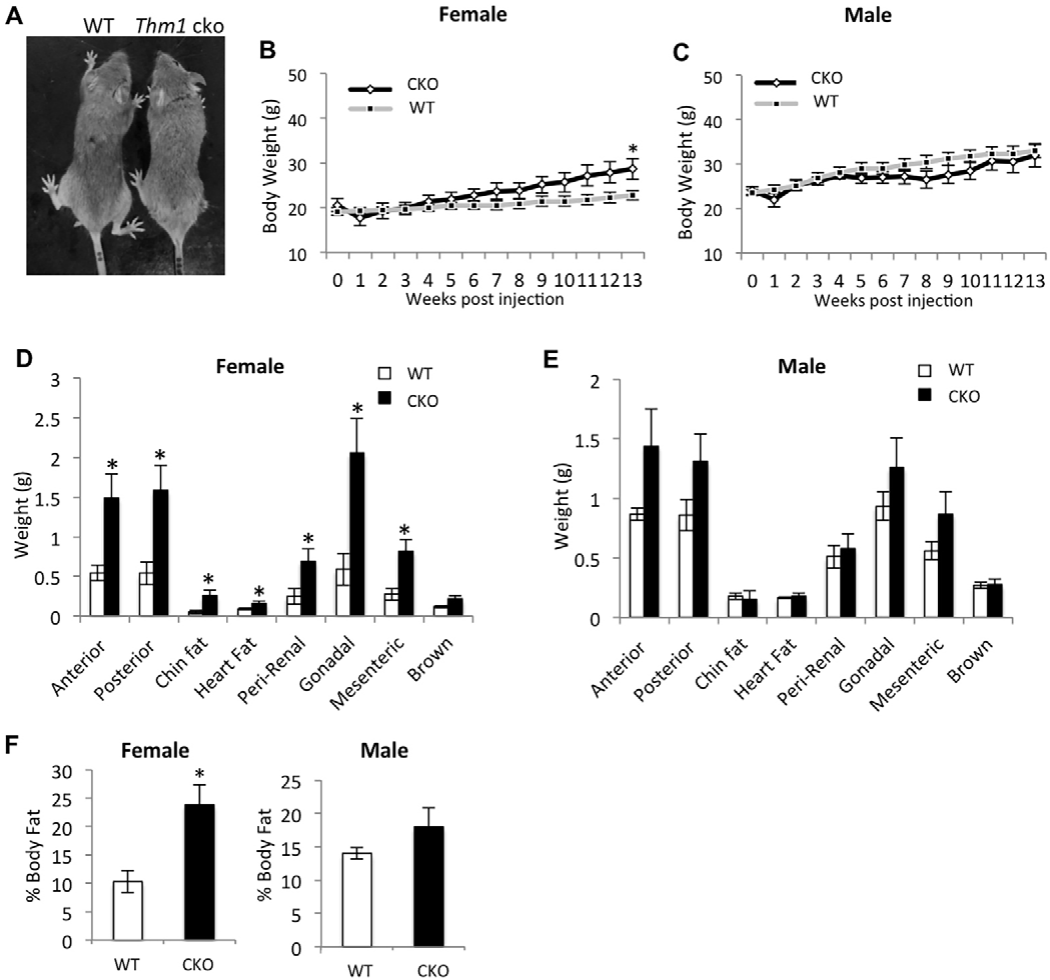
**Hyperphagia is a primary cause of obesity in *Thm1*-cko mice.** (A) Pair-fed WT and *Thm1*-cko mice 13 weeks post-tamoxifen injection. (B,C) Weekly body weights of pair-fed mice over a 13-week period. Data points represent means±s.e.m. (D,E) Adipose tissue weights of pair-fed mice at 13 weeks post-tamoxifen injection. Error bars represent means±s.e.m. Two-tailed unequal variance *t*-test. *P<0.05. (F) Percent body fat of pair-fed mice at 13 weeks post-tamoxifen injection. Error bars represent means±s.e.m. *P<0.05; *N*=7 WT females; *N*=7 *Thm1-cko* females; *N*=6 WT males; *N*=8 *Thm1-cko* males.

To determine the cause of weight gain in *Thm1*-cko mice, we examined energy expenditure and food intake. Using a force plate actimeter, activity of WT and *Thm1*-cko mice were monitored at mid-day during 10-min intervals 1 week, 6 weeks and 13 weeks following gene deletion. Total distance traveled, rate of movement, and focused stereotypy (repetitive movement) were not significantly different between WT and *Thm1*-cko mice (Fig. S4). In contrast, measurements of daily food intake over the 13-week period revealed that female and male *Thm1*-cko mice consumed ∼19% and 9% more diet than female and male WT mice, respectively. To determine whether hyperphagia caused the *Thm1*-cko weight gain, we pair-fed control and *Thm1*-cko mice for 13 weeks following *Thm1* ablation. Throughout these 13 weeks, WT and *Thm1*-cko mice showed similar body weights (Fig. 4A-C), suggesting hyperphagia is a main contributor to the *Thm1*-cko obese phenotype. At the 13-week time point, we noted that pair-fed *Thm1*-cko females did emerge slightly heavier than control females (Fig. 4B), with heavier white adipose tissues (Fig. 4D) and ∼2.3-fold higher percent body fat than control females (Fig. 4F). Pair-fed *Thm1*-cko male mice did not exhibit elevated weight gain relative to control males (Fig. 4C,E,F). These findings suggest an increased propensity in *Thm1*-cko females to increase adipose tissue weight.

**Fig. 5.**
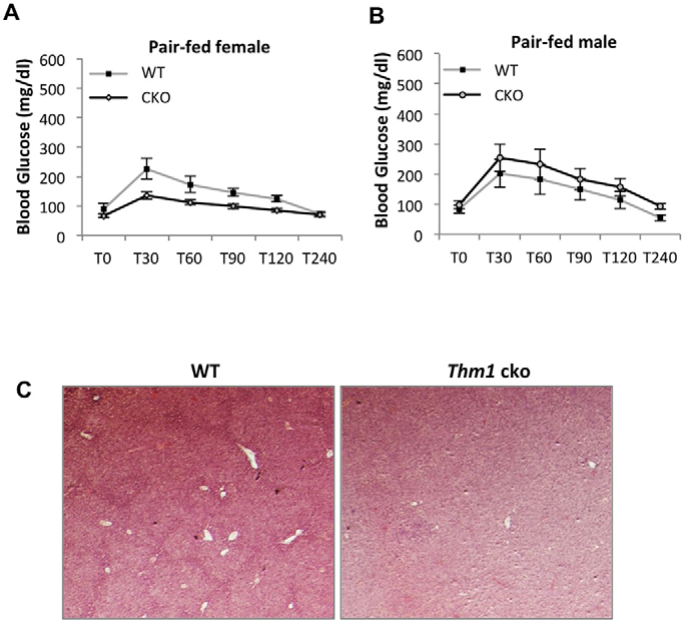
**Pair-fed *Thm1*-cko mice show normal glucose metabolism and liver morphology.** (A,B) Glucose tolerance test for pair-fed mice at 13 to 20 weeks post-*Thm1* deletion. *N*=4 WT; *N*≥4 *Thm1*-cko each for male and female. Data points represent means±s.e.m. Two-tailed unequal variance *t*-test. (C) H&E staining of liver sections from WT and *Thm1*-cko mice at 13 weeks post-tamoxifen injection.

In pair-fed *Thm1*-cko mice, blood glucose levels remained similar to those of WT mice at 0, 6 and 13 weeks post-*Thm1* deletion. In response to a GTT, pair-fed female and male WT and *Thm1*-cko mice showed similar glucose clearance (Fig. 5A,B). Additionally, organ weights, including that of liver, were similar between pair-fed WT and *Thm1*-cko mice (Fig. S5). Histological analysis revealed normal liver morphology in pair-fed *Thm1*-cko mice (Fig. 5C). These data indicate that the DM2 and fatty liver disease present in *ad libitum*-fed *Thm1*-cko mice were consequences of the obese phenotype.

### *Thm1*-cko mice exhibit altered neuropeptide gene expression in the hypothalamic arcuate nucleus

**Fig. 6.**
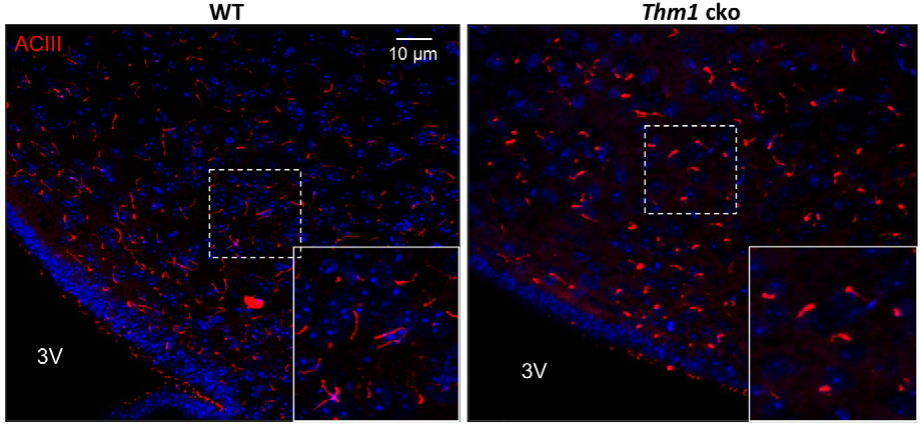
***Thm1*-cko primary cilia are stunted with a bulbous distal tip and show enrichment of AC3.** Immunostaining for AC3 (ACIII) on sections of the arcuate nucleus (ARC). Inset shows higher magnification of dotted region of the ARC. 3V, third ventricle.

**Fig. 7.**
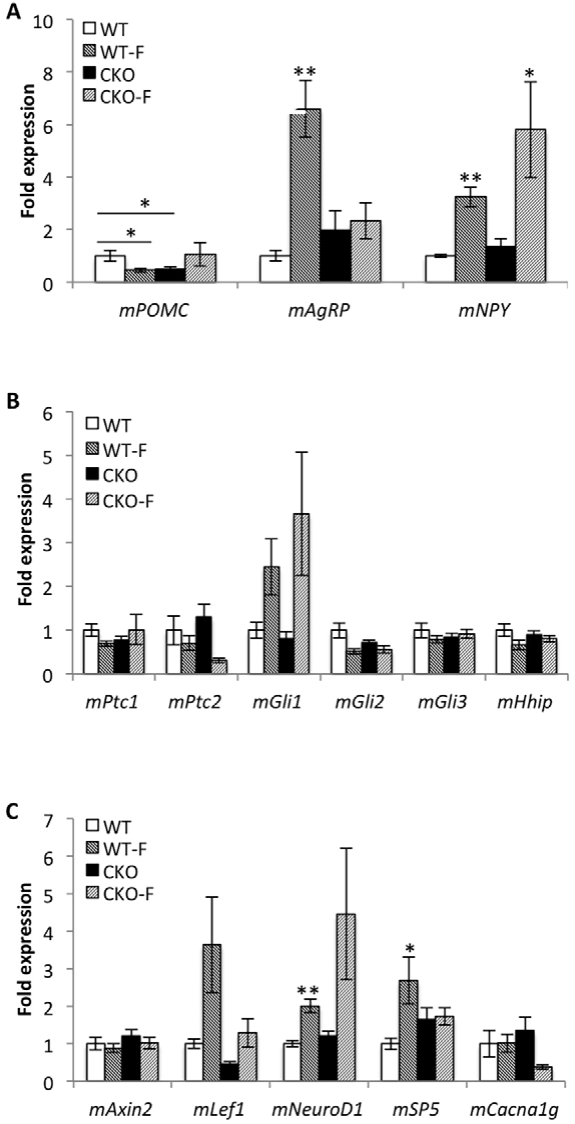
**Pre-obese *Thm1*-cko mice exhibit altered neuropeptide gene expression in the hypothalamic arcuate nucleus.** Gene expression analysis at 2 weeks post-*Thm1* deletion on extracts of the arcuate nucleus using qPCR for (A) appetite-regulating neuropeptides, and (B) hedgehog and (C) Wnt target genes. Gene expression was normalized to β-actin expression. Error bars represent means±s.e.m. Two-tailed unequal variance *t*-test. *P≤0.05; **P≤0.005; *N*≥7 WT; *N*≥7 *Thm1*-cko each for fasted (F) and non-fasted mice.

In response to signals such as leptin or insulin, *POMC*- and *AgRP/NPY*-expressing neurons of the ARC regulate feeding response (Belgardt et al., 2009). A satiated state causes upregulation of *POMC* expression, which attenuates food-seeking behavior and increases physical activity. Conversely, fasting increases *AgRP* and *NPY* expression, which augments food-seeking behavior and decreases physical activity. To determine if *Thm1*-cko mice exhibit signaling defects in the ARC, we examined primary cilia and neuropeptide gene expression in the ARC two weeks following gene deletion. At this time point, WT and *Thm1*-cko body weights were similar (Fig. 1B,C), so that any observed differences in gene expression between WT and *Thm1*-cko mice might suggest mechanisms that initiate the obese phenotype. To examine primary cilia, sections of the hypothalamus were immunostained for the neural ciliary marker, adenylyl cyclase 3 (AC3, also known as ADCY3) (Bishop et al., 2007). The ARC is situated around the ventrolateral region of the third ventricle. Neuronal primary cilia in this region in *Thm1*-cko mice were shortened with a bulbous distal tip and showed more intense expression of AC3, suggesting an accumulation of proteins in the mutant cilia (Fig. 6). Quantitative PCR of RNA lysates from the ARC of WT and *Thm1*-cko mice revealed ∼50% lower *POMC* expression in *Thm1*-cko mice than in WT mice (Fig. 7A). Because POMC neuropeptides attenuate food-seeking behavior, this decrease in *POMC* might be a primary cause of the hyperphagia. In response to fasting, WT ARC extracts showed decreased *POMC* expression and increased *AgRP* and *NPY* expression. In contrast, fasting of *Thm1*-cko mice increased *NPY* transcript levels, but did not cause expected alterations of *POMC* and *AgRP* transcripts. These results suggest that loss of *Thm1* causes misregulation of the feeding/activity signaling axis in the ARC.

Primary cilia regulate hedgehog (Hh) and Wnt signaling pathways (Nozawa et al., 2013; Oh and Katsanis, 2013), and these pathways have been implicated in various aspects of adipose biology (Christodoulides et al., 2009; Cousin et al., 2007). Activation of Hh or Wnt signaling inhibits adipocyte differentiation (Ross et al., 2000; Spinella-Jaegle et al., 2001), and activation of Wnt signaling in mature brown adipocytes drives their conversion to white adipocytes (Tseng et al., 2005). Yet a role for these developmental signaling pathways in regulating energy homeostasis in the hypothalamus has not been investigated. Previously, we identified THM1 as a negative regulator of Hh signaling during embryonic development, including in neuronal tissue (Tran et al., 2008). Thus, we queried whether Hh signaling was altered in the ARC of *Thm1*-cko mice two weeks following gene deletion. Using qPCR, we examined levels of Hh targets and signaling components, *Patched1* (*Ptc1*), *Ptc2*, *Gli1*, *Gli2*, *Gli3* and *Hhip*. In both non-fasted and fasted states, we did not observe significant differences in gene expression between WT and *Thm1*-cko mice (Fig. 7B).

Because mouse embryonic fibroblasts lacking *Thm1* show an increased response to Wnt3a ligand (Tran et al., 2014), we further examined expression of neural Wnt signaling targets *Axin2*, *Lef1*, *NeuroD1*, *SP5* and *Cacna1g* in the ARC of *Thm1*-cko mice. In non-fasted states, gene expression levels between WT and *Thm1*-cko mice were similar (Fig. 7C). In response to fasting, *SP5* and *NeuroD1* expression increased in WT mice, suggesting canonical Wnt signaling might be involved in the fasting response. In contrast, fasting of *Thm1*-cko mice did not significantly increase *SP5* expression (*P*=0.088) nor alter *NeuroD1* expression, supporting an aberrant response to fasting in *Thm1*-cko mice.

In addition, we examined serum levels of leptin, insulin, C-peptide, resistin and GIP in *Thm1*-cko mice at this time point; two weeks following gene deletion and prior to weight gain. Levels of all metabolites were similar between WT and *Thm1*-cko mice (Fig. S6), indicating that the metabolic disturbances observed in obese *Thm1*-cko mice follow the molecular changes that occur in the ARC.

## DISCUSSION

In this study, we demonstrate that deletion of *Thm1*, a component of the IFT-A complex, causes hyperphagia-induced obesity in mice. These findings add a new mutant class to the list of ciliary mouse models of hyperphagia and obesity, which includes mutants of the BBS complex, of *Alms1*, of the IFT-B complex, and of the transition zone (Arsov et al., 2006; Collin et al., 2005; Davenport et al., 2007; Rahmouni et al., 2008; Stratigopoulos et al., 2014). The *Thm1*-cko obese phenotype further causes glucose intolerance and insulin resistance, which together indicate metabolic syndrome. In the human population, metabolic syndrome has become epidemic worldwide and a predictor of DM2, cardiovascular disease, and non-alcoholic fatty liver disease (Shin et al., 2013). Obese *Thm1*-cko mice also develop diabetes and fatty liver disease, modeling the human condition.

Prior to the increased weight gain and elevated serum metabolite levels in *Thm1*-cko mice, *POMC* mRNA levels in the ARC were reduced. This *POMC* reduction is likely a main cause of the hyperphagia. *POMC*-derived peptides attenuate food-seeking behavior and *POMC*-null mice are hyperphagic and obese (Yaswen et al., 1999). Further, deletion of *Ift88* in *POMC*-expressing cells in mice resulted in hyperphagia and obesity, demonstrating that ciliary function specifically of *POMC*-expressing neurons is crucial to the neuronal circuitry that controls appetite (Davenport et al., 2007). Reduced *POMC* levels have been reported also in obese *Bbs2^−/−^*, *Bbs4^−/−^* and *Bbs6^−/−^* mice (Rahmouni et al., 2008), in obese *Rpgrip1l^+/−^* mutant mice (Stratigopoulos et al., 2014), and recently, in pre-obese *Bbs1;Cre^LR^^b^* cko mice, in which *Bbs1* was deleted in leptin receptor (LRb)-expressing cells (Guo et al., 2016). In contrast, *POMC* levels were not reduced in *Ift88;Cre^LRb^* cko mice. These data suggest that deficiency of *Bbs*, *Rpgrip1l* and *Thm1* might perturb similar signaling pathways.

In *Bbs2^−/−^* and *Bbs6^−/−^* mutant mice, *POMC*-expressing neurons were decreased by ∼20%, contributing to but not completely accounting for the 40% reduction in *POMC* transcript levels (Seo et al., 2009). Data in our lab show that the number of P-STAT3-expressing neurons, which comprise both *POMC*- and *AgRP*-expressing neurons (Ha et al., 2013), is similar between WT and *Thm1*-cko mice, suggesting that the number of these neuronal populations is unaffected. This is likely because *Thm1* was deleted in adulthood, whereas *Bbs2* and *Bbs4* were deleted from the beginning of embryogenesis. Thus, we propose that the decrease in *POMC* transcripts in *Thm1*-cko mice results from altered signaling and not from POMC neuron number. Deregulated *POMC* transcription in *Thm1*-cko mice in response to fasting further supports altered signaling. The ciliary phenotypes of these mutants differ; cilia are ablated in complex B *Ift88* and *Kif3a* mutants (Davenport et al., 2007), unchanged at the structural level in *Bbs2* and *Bbs4* mutants (Berbari et al., 2008), shortened with a bulb-like structure at the distal tip in IFT complex A *Thm1* mutants (Tran et al., 2008) and lengthened in transition zone *Rpgrip1l* mutants (Gerhardt et al., 2015; Stratigopoulos et al., 2014). Collectively, these data suggest that regulation of *POMC* and of molecular components of *POMC*-expressing cells constitutes an important underlying mechanism of obesity in these ciliary mutants.

In WT mice, fasting decreased *POMC* expression and increased *AgRP* and *NPY* expression to increase food-seeking behavior. In contrast, in *Thm1*-cko mice, fasting caused upregulation of *NPY*, whereas *POMC* and *AgRP* levels remained unchanged, suggesting that the *Thm1* ciliary defect impairs the sensing of changes in peripheral signals. Similar to *Thm1*-cko mice, fasted *Bbs2^−/−^*, *Bbs4^−/−^* and *Rpgrip1l^+/−^* mice showed an impaired *POMC* transcriptional response, whereas in contrast to *Thm1*-cko mice, *AgRP* and *NPY* transcriptional responses were normal (Seo et al., 2009). Our findings in the *Thm1*-cko mice reflect differential regulation of *NPY* and *AgRP*. Differential regulation of *NPY* and *AgRP* has also been observed in rats devoid of a functional leptin receptor (Korner et al., 2001) and in fasted C57BL/6 mice that upon re-feeding showed restored *POMC* levels and reduced *NPY* levels, but unaltered *AgRP* expression (Swart et al., 2002).

Although leptin regulates *POMC*, *NPY* and *AgRP* expression, the role of primary cilia in leptin signaling has been controversial. Genetic deletion of *Bbs2*, *Bbs4*, *Bbs6* or of *Rpgrip1l*, which traffic proteins to the cilium, and siRNA-mediated knock-down of *Kif3a* and *Ift88* in the hypothalamic third ventricle of mice, impaired response of these animals to the appetite- and weight-reducing effects of exogenous leptin (Han et al., 2014; Seo et al., 2009; Stratigopoulos et al., 2014). These studies were performed on lean, calorie-restricted or pre-obese mice that were not hyperleptinemic, suggesting ciliary dysfunction causes a primary defect in leptin signaling. In contrast, pre-obese *Bbs4-null* mice and both pre-obese and lean, calorie-restricted mice with a global *Ift88* deficiency induced during adulthood responded normally to an intraperitoneal injection of leptin, suggesting leptin resistance is a secondary consequence of the obese phenotype (Berbari et al., 2013). A recent report might account for some of these discrepancies. *Bbs1;Cre^LRb^* mice developed morbid obesity as a result of hyperphagia and reduced energy expenditure, in contrast to *Ift88;Cre^LRb^* mice, which showed mild weight gain that was not a result of hyperphagia (Guo et al., 2016). Further, calorie-restricted *Bbs1;Cre^LRb^* mice showed leptin resistance, whereas *Ift88;Cre^LRb^* mice did not. Knock-down of *Bbs1*, but not of *Ift88*, showed impaired trafficking of the leptin receptor to the cell membrane *in vitro*, suggesting that BBS1, but not IFT88, is involved in intracellular trafficking of the leptin receptor. These results further imply that BBS leptin resistance is independent of the primary cilium. The results of these collective studies underscore the need to investigate leptin sensitivity in other ciliary mouse mutants, including those of a different ciliary mutant class, like *Thm1*.

In addition to leptin, other signals such as insulin, glucose and estrogen, regulate *POMC*, *NPY* and *AgRP* expression (Gao et al., 2007; Varela and Horvath, 2012). Six hours of re-feeding following a 24-h fast in C57BL/6 mice restored *POMC* and *NPY* levels, but leptin administration at a dose that reduced food intake did not affect neuropeptide gene expression, suggesting that multiple signals work in concert to effect changes in *POMC*, *AgRP* and *NPY* (Swart et al., 2002). It has been shown that during embryonic development, primary cilia regulate multiple signaling pathways. Similarly, it is possible that multiple ciliary-mediated pathways converge on neuropeptide gene expression. Identification of these signals and pathways will be crucial to delineating the role of primary cilia in energy homeostasis.

The importance of primary cilia during development is well recognized, but the role of primary cilia during tissue maintenance is only beginning to be understood. This study and others (Berbari et al., 2013; Davenport et al., 2007) demonstrate that primary cilia are important in adult hypothalamic neurons. This contrasts with the kidney, where loss of primary cilia during kidney development causes aggressive cystic kidney disease, but loss of primary cilia once the kidney has matured results in very slow, late-onset cyst development initiating 6 months following gene deletion (Davenport et al., 2007). Consistent with this, *Thm1*-cko kidneys showed a normal phenotype at the end of this study period 13 weeks following *Thm1* deletion at 5 weeks of age (data not shown). The sensitivity of the adult hypothalamus to ciliary changes might be attributed to the plasticity required for regulating energy homeostasis (Horvath, 2005).

We observed phenotypic differences between *Thm1*-cko females and males. *Thm1*-cko females showed a greater increase in adipose tissue weight, which was reflected also in higher levels of leptin and resistin. Similarly, *Bbs4^−/−^* and *Bbs1^−/−^* female mice show a more severe obese phenotype than their male counterparts (Eichers et al., 2006; Guo et al., 2016). Yet *Thm1*-cko males, and not females, were diabetic at the end of the study period. Such differences are consistent with a study that showed gender-specific metabolic changes in *ob/ob* mice, with changes in *ob/ob* males and females associated with insulin signaling and lipid metabolism, respectively (Won et al., 2013). Additionally, pair-fed *Thm1*-cko females, but not pair-fed *Thm1*-cko males, were heavier than control littermates at the end of the 13-week study, further showing an increased tendency in *Thm1*-cko females to increase adipose tissue weight. Pair-feeding of *Bbs* mutant mice similarly did not completely rescue the increase in fat depots relative to control littermates, suggesting reduced energy expenditure (Guo et al., 2016; Rahmouni et al., 2008). Similarly, energy expenditure such as in the form of basal metabolic rate or thermoregulation, which could not be measured by the actimeter, might also be affected by deficiency of *Thm1*.

Finally, loss of THM1 resulted in shortened primary cilia with bulbous distal tips and increased ciliary localization of AC3. This phenotype, including protein accumulation in the mutant cilia, is characteristic of an IFT-A mutant phenotype (Tran et al., 2008). Yet this enrichment of AC3 in *Thm1*-mutant cilia contrasts with the absent or decreased ciliary localization of AC3 in mouse embryonic fibroblasts of other complex A mutants, *Ift144* and *Ift122* (Liem et al., 2012) and also of transition zone mutants, *Rpgrip1l* (Stratigopoulos et al., 2014). Deletion of *AC3* in mice causes obesity (Wang et al., 2009), whereas a gain-of-function mutation in *AC3* protects mice from diet-induced obesity (Pitman et al., 2014). The contrasting effects of *Thm1* deficiency versus *Ift144* or *Ift122* deficiency on AC3 ciliary localization might reflect the unique biochemical functions of individual IFT proteins. Further, the increased ciliary localization of AC3 in *Thm1*-cko neuronal cilia coupled with the obese phenotype questions the functionality of the adenylyl cyclase, and implicates a role for THM1 in regulating AC3 function.

In summary, the *Thm1*-cko mouse provides the first IFT complex A mouse model of hyperphagia and obesity. Prior to increased weight gain, deficiency of *Thm1* downregulates *POMC* expression in the ARC and misregulates *POMC* and *AgRP* expression in response to fasting. Interestingly, the *Thm1*-mutant phenotype suggests mechanisms similar to *Bbs* and transition zone mutants and not to *Ift-B* mutants, suggesting differential roles of IFT-B and IFT-A complexes in regulating energy homeostasis. Future investigations into the molecular mechanisms regulating neuropeptide gene expression and the role of THM1 and primary cilia in different neuronal populations of the ARC will provide a greater understanding of the neural circuitry that controls energy homeostasis, which will offer potential therapeutic targets against hyperphagia and obesity.

## MATERIALS AND METHODS

### Mice

*Thm1*-cko mice were generated as previously described (Tran et al., 2014). Briefly, *Thm1*-cko mice were generated with one allele harboring the *aln* mutation, which results in a null allele, and a floxed allele containing LoxP sites flanking exon 4. Deletion of the floxed exon was carried out using tamoxifen-inducible *ROSA26CreERT* mice (Jackson Laboratories, Stock 004847). Cre recombinase expression was induced at 5 weeks of age by i.p. injection of 10 mg tamoxifen/40* *g mouse weight. Tamoxifen (Sigma T5648) was suspended in corn oil (Sigma C8267) at 30 mg/ml. To examine recombination in control (*Thm1^fl/+^;CreERT+*) or *Thm1*-cko (*Thm1^fl/aln^;CreERT+*) mice, Gt(ROSA)26Sor^tm4(ACTB-tdTomato,-EGFP)Luo^/Gt(ROSA)26Sor^tm4(ACTB-tdTomato,-EGFP)Luo^ reporter mice (Jackson Laboratories, Stock 007676), which express tdTomato in non-recombined tissue and EGFP in recombined tissue, were bred into the *Thm1^fl/fl^* lines. *Thm1^fl/fl^;tdTom+* females were then bred with *Thm1^aln/+^;CreERT+* males to generate progeny expressing the reporter. Tissue from *Thm1^fl/+^;tdTom+* or *Thm1^fl/aln^;tdTom+* mice and from *Thm1^fl/+^;CreERT+;tdTom+* or *Thm1^fl/aln^;CreERT+;tdTom+* were embedded in OCT compound, cryosectioned at 10-µm thicknesses, and viewed under a fluorescence microscope. Mice were on a mixed genetic background containing FVB, SV129 and C57BL/6J strains. All animal procedures were conducted in accordance with KUMC-IACUC and AAALAC rules and regulations.

### Feeding

Mice were fed a Breeder diet (PicoLab 5058). To measure daily diet consumption, WT and *Thm1*-cko mice were housed individually. Pair feeding was performed by monitoring the daily diet intake (by weight) of control mice and providing the same amount to *Thm1*-cko littermates.

### Serum collection

Submandibular blood (100-200 µl) was collected in a Microvette CB300z blood collection tube (Kent Scientific, Torrington, CN). Serum was isolated by centrifugation for 10 min at 1800×***g*** at 4°C using a tabletop centrifuge (PrismR; C2500-R). Serum was collected and treated with protease inhibitor cocktail (Sigma) and stored at −80°C until analysis. Serum was analyzed using a Milliplex MAP Mouse Metabolic Hormone Magnetic Bead Panel – Metabolism Multiplex assay (EMD Millipore; MMHMAG-44K).

### Activity levels

Activity levels were monitored by placing the mice onto a BASi Force Plate Actimeter (KUMC Rodent Behavior Facility) that uses force transduction to monitor and track locomotor activity. Mice were allowed free access to the 1 m×1 m actimeter arena for 10 min during the light cycle. Force Plate Actimeter activity software reports locomotion behaviors including distance traveled, rate of movement, and focused stereotypy.

### Glucose tolerance test

WT and *Thm1*-cko mice were fasted for 8-12 h prior to determining the baseline glucose level. A bolus (2 mg/kg) of 20% glucose in PBS solution was delivered via i.p. injection at time 0 (T0). Blood glucose level was determined using a Contour blood glucose monitor (Model 7151H, Bayer Corp.) and was sampled from the tail vein at 30-min intervals for 4 h. Glucose levels between WT and *Thm1*-cko mice were compared using a Students' *t*-test at each time interval.

### qPCR of ARC RNA extracts

Whole mouse brain was harvested, then further dissected to obtain the whole hypothalamus, and finally, the arcuate nucleus was isolated from the hypothalamus. Isolated tissues were immediately placed onto dry ice for storage. Tissue was homogenized with a pestle (www.bioexpress.com; C-3260-1) and RNA was extracted using the RNeasy mini kit (Qiagen). cDNA was generated using qScript cDNA Supermix (Quanta Biosciences, 95048-500) and real-time PCR was performed using qPCR PerfeCTa SYBR Green FastMix (Quanta BioSciences, 95072-012) in a BioRad CFX Connect Real-Time System. Intron-spanning primers for qPCR assays were designed using the Roche Universal ProbeLibrary Assay Design Center and synthesized by IDT Technologies. All primer sequences are listed in Table S1.

### Tissue processing

Mice were anesthetized with an i.p. injection of ketamine and xylazine, and were cardiac perfused. Perfusion was performed with 7 ml of phosphate buffer saline (PBS) followed by 7 ml of 4% paraformaldehyde (PFA) in PBS at ∼3.5 ml/min. Tissues were isolated and submersed in 4% PFA on ice for 2-12 h or in Bouin's fixative (Poly Scientific, Bay Shore, NY) for >24 h. Bouin's fixed tissues were dehydrated through an ethanol series, paraffin-embedded and sectioned at 7-µm thicknesses. Brain tissues were fixed in 4% PFA, infused with 30% sucrose, embedded in Tissue-Tek OCT compound (Sakura) and cryosectioned at 10-15-µm thicknesses.

### Histology

Tissues were stained with hematoxylin and eosin using a standard protocol. Liver tissues were stained using Oil Red O (ORO) stain (Sigma-Aldrich, O0625). An initial stock of 0.5% ORO in 2-propanol was diluted to 0.3% ORO in 60% isopropanol and filtered into a Coplin jar for staining. PFA-fixed sections were rinsed once with 60% isopropanol then immersed in 0.3% ORO in 60% isopropanol for 12 min. Sections were rinsed with 60% isopropanol for 3-4 min to remove excess ORO. Nuclei were stained with hematoxylin solution (5 dips) and rinsed with distilled H_2_O for 3 min. Sections were mounted with 100 µl ImmunoHistoMount solution (Sigma, I1161). Staining was viewed and imaged using a Nikon 80i microscope equipped with a Nikon DS-Fi1 camera.

### Immunofluorescence

Fixed tissues were OCT-embedded and sectioned at 10-15 µm. Primary antibodies were diluted into blocking buffer (2% BSA in PBS) and tissue sections were incubated at 4°C for 1-12 h. Primary antibody used was anti-adenylate cyclase III (C-20; Santa Cruz, sc-588; 1:100 dilution) and secondary antibody used was Alexa Fluor 594 goat anti-rabbit (Thermo Fisher, A-11012; 1:1000 dilution). Tissues were labeled with DAPI (5 µg/ml in PBS) to visualize nuclei. Tissue sections were then mounted with Fluoromount-G mounting media (Electron Microscopy Services). Immunolabeled tissues were viewed and imaged using a Leica TCS SPE confocal microscope configured on a DM550 Q upright microscope.

### Statistics

Two-tailed unequal variance *t*-tests were performed. *P*-value <0.5 was considered significant.
